# Supplementary material for: Distinguishing ChatGPT(-3.5, -4)-generated and human-written papers through Japanese stylometric analysis
Source: PLoS One. 2023 Aug 9;18(8):e0288453. doi: 10.1371/journal.pone.0288453 (PMC10411719; doi:10.1371/journal.pone.0288453)
Supplement: Attachment — Submitted filename: Response to Reviewers.docx [file pone.0288453.s006.docx]

Answer and Response to Editor and Reviewer 1 comments:

Thank you for your constructive review.

I revised several points along with your comments.

Please confirm revised paper.

Thank you.

1.”Bing by Microsoft”(p4.l78, p16.l371)

I modified “Bing” to “Bing AI”, along with reviewer’s comment.

2. Detailed explain: “Stylometric features” and “variables” (p8,l171)

I more politely explained “Stylometric features”, “variables”, “POS tagger” in this paper.

3. revised representation (p9,l206)

I replaced “this” to “that”, along with reviewer’s comment.

4. Increasing parameters and future (p15,l344)

I revised some points, for example, “AI-generated” to “ChatGPT-generated” (Thus, even if the number of parameters increases in the future, the distribution of ChatGPT-generated texts may not be close to that generated by humans in each stylometric feature.).

But I think above claim is warranted, because this study showed evidence about relationship between difference of the number of parameters and no difference of stylometric features. So I used the expression 'may' because it is weak evidence.

5. Deleted “LINE”

LINE is a kind of SNS mainly used in the Asian, but not around the world. So I deleted this word.

6. English Native check

I requested an English proofreader.

7. revised representation

I replaced “text-generated AI” to “chatbots”.

Answer and Response to Reviewer 1 comments:

1. Revised explanation of GPT technology in Introduction(p4,l71)

I revised this paragraph in whole.

2. Additional analysis for cross validation(p12, l274)

The number of K is too fine, it is not correct, because the sample size in this study is small. Therefore, I conducted “10-fold” cross validation.

3. Adding explain(p7, l155)

I added explain of how to make texts (1,000 characters), along with reviewer’s comment.

4. Adding explain

I explained hyperparameters for RF and used software and packages, along with reviewer’s comment.

Answer and Response to Reviewer 2 comments:

1. Adding reference about previous researches

I added and wrote more references including state-of-the-art references.
