# Supplementary material for: Distinguishing ChatGPT(-3.5, -4)-generated and human-written papers through Japanese stylometric analysis
Source: PLoS One. 2023 Aug 9;18(8):e0288453. doi: 10.1371/journal.pone.0288453 (PMC10411719; doi:10.1371/journal.pone.0288453)
Supplement: Attachment — Submitted filename: Response to Reviewers.docx [file pone.0288453.s008.docx]

Answer and Response to Editor:

Thank you for your constructive review.

We mostly revised grammar along with your whole comments.

In addition to grammar revision, we added explanation about confusion matrix and each performance metric (accuracy, recall, precision, and f1 score) along with your comments.

Moreover, from the last time submission, we found new paper and added this paper.

Please confirm revised paper.

Thank you.
